# An Analysis of the Usage of Retinal Imaging Technology in the Detection of Age-Related Macular Degeneration

**DOI:** 10.7759/cureus.40527

**Published:** 2023-06-16

**Authors:** Parsa Riazi Esfahani, Akshay J Reddy, Jack Thomas, Dillon A Sommer, Anna Nguyen, Victoria Farasat, Neel Nawathey, Alex Bachir, Telak Brahmbhatt, Rakesh Patel

**Affiliations:** 1 Department of Medicine, California University of Science and Medicine, Colton, USA; 2 Department of Biology, Irvine Valley College, Irvine, USA; 3 Department of Health Sciences, California Northstate University, Rancho Cordova, USA; 4 Department of Medicine, Geisinger Commonwealth School of Medicine, Scranton, USA; 5 Department of Internal Medicine, East Tennessee State University Quillen College of Medicine, Johnson City, USA

**Keywords:** fundus fluorescein angiography (ffa), fa, cfp, oct (optical coherence tomography), armd

## Abstract

Age-related macular degeneration (AMD) is a disease that worsens the central vision of numerous individuals across the globe. Ensuring that patients are diagnosed accurately and that their symptoms are carefully monitored is essential to ensure that adequate care is delivered. To accomplish this objective, retinal imaging technology is necessary to assess the pathophysiology that is required to give an accurate diagnosis of AMD. The purpose of this review is to assess the ability of various retinal imaging technologies such as optical coherence tomography (OCT), color fundus retinal photography, fluorescein angiography, and fundus photography. The statistical methods that were conducted yielded results that suggested that using OCT in conjunction with other imaging technologies results in a higher detection of symptoms among patients that have AMD. Further investigation should be conducted to ascertain the validity of the conclusions that were stated within the review.

## Introduction and background

Age-related macular degeneration (AMD) is a chronic illness that affects 196 million people globally and damages the central retina. As the condition advances, it causes the loss of the visual field [1]. Currently, the standard within health care is to use optical coherence tomography (OCT) imaging to monitor the progression of AMD in patients [1-3]. The signs of this condition include visual abnormalities and impaired central vision [1]. In the later stages of the disease, neovascularization and geographic atrophy typically cause visual impairment [2]. AMD is a multifaceted illness involving a complicated interaction between aging, environmental risk factors, and genetic predisposition. Strongly implicated in AMD etiology are chronic inflammation, lipid accumulation, tobacco usage, oxidative stress, and poor extracellular matrix preservation. Therefore, AMD can be divided into exudative (wet) and non-exudative subtypes (dry), with the dry subtype being more common. When the choroidal neovascular membranes behind the retina leak fluid and blood, wet AMD arises. If wet AMD is diagnosed in its early stages, anti-vascular endothelial growth factor (A-VEGF) injections can be used to treat it [3]. This, in turn, causes retinal damage. Dry AMD is characterized by degeneration of the retina and choroid due to thinning or detachment of the retinal pigment epithelium (RPE) [3]. Clinical examination and investigations are used to provide an accurate diagnosis. Modern ocular imaging tools make it possible to quantify changes in the RPE, photoreceptors, and choriocapillaris as the disease advances [4,5]. When coupled with the known histology of AMD, these data may give meaningful measures of therapy efficacy that are more accurate and consistent than traditional endpoints like visual acuity and rate of geographic atrophy expansion. The objective of this review is to examine how various imaging modalities can be utilized to detect AMD.

## Review

Methods

**Figure 1:**
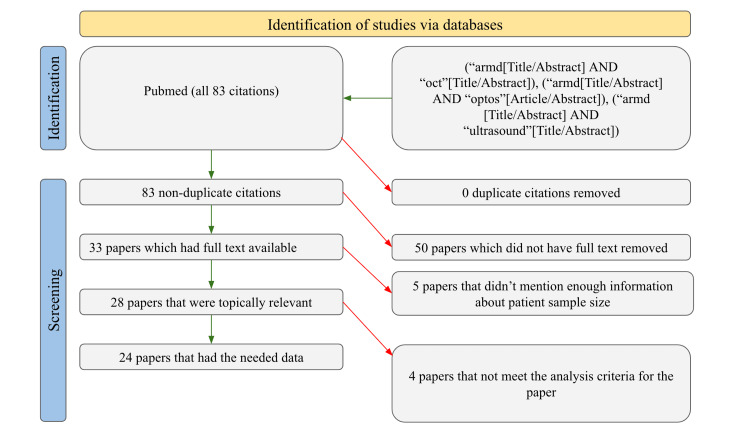
PRISMA Article Filtration Diagram PRISMA, Preferred Reporting Items for Systematic Reviews and Meta-Analyses.

The PubMed database was searched for articles on the use of imaging technologies to detect AMD. All time periods were searched using the exact search terms "armd and oct," "armd and optos," and "armd and ultrasound." This resulted in a total of 83 papers, of which 83 were unique and not duplicates, 33 had full texts available, 28 were topically relevant, and only 24 met the analysis requirements for our evaluation. The information gathered from these 24 investigations included the imaging technologies used, the vascular and non-vascular ocular disorders discovered in patients, the patient's past medical conditions, and the sample size. Excluded were studies that lacked sufficient data to overlap with at least two of these categories. This was done to eliminate personal bias. The authors of this report provided a more detailed illustration of the filtration process in Figure 1.

Imaging technology 

Retinal imaging has undergone a revolution in the last 10 years, giving patients with AMD precise views of retinal abnormalities, including that of AMD [5]. This assists in discovering the various retinal imaging techniques and how they aid ophthalmologists in the detection and treatment of AMD. Optics, cameras, computers, and software developments have led to the development of new retinal imaging techniques, which have shown to be very useful as diagnostic and therapeutic tools for AMD [6-10]. In terms of retinal imaging, OCT has made the biggest strides [6,8]. This method creates a cross-sectional view of the retina on a computer screen using infrared light reflections off the retina. The images are captured quickly and without the use of any jarring strong light flashes. In patients with wet AMD, they reveal fluid pockets in the retina and choroidal neovascularization, which can be used to determine how frequently anti-VEGF injections should be administered [7,10]. Additionally, they exhibit retinal thinning brought on by cell death in individuals with late dry AMD as well as drusen, which are tiny deposits under the retina in early dry AMD patients (also called geographic atrophy). An effective imaging technique for determining the existence, location, and size of the neovascular complex, which is made up of the choroidal neovascular lesion and its accompanying components, is fluorescein angiography (FA) [6-10]. Fluorescein, a yellowish dye, is injected into a vein, commonly in your arm. The dye needs 10 to 15 seconds to circulate throughout the body. The eye's blood vessels are then exposed to the dye, making them fluoresce. Once this is complete, a unique camera is utilized to obtain images that are captured while the dye travels across the retina [11-14]. A diagnosis can be determined using these images. FA also yields critical physiologic information (leakage, etc) that may not be picked up on other testing modalities [9]. FA characterization is utilized extensively in AMD clinical trials for both eligibility and treatment outcomes. Upon comparing signs detected by OCT compared to OCT and other imaging technology, it was found that there is a significant difference between the number of signs that are detected when OCT was used alongside other imaging technologies (p-value = 0.0395, t-value = 2.1960). These values were obtained from a statistical analysis that was performed using data collected from various studies within this review. This indicates that using OCT with other imaging technologies will yield a better diagnosis and, ultimately, better treatments for the disease. Though these values provide insight into the better diagnosis of AMD, the results might be skewed due to the small sample size that was used. Therefore, further research on image technologies and AMD diagnosis should be done. 

Associated pathophysiology

According to the data presented in Table 1, the most common vascular sign that was associated with the diagnosis of AMD was neovascularization [6-29]. This sign was mentioned as a very significant occurrence within 24 of the studies that were analyzed in the review. Neovascularization, the growth of new blood vessels, is a result of the excess production of VEGF by the retina when patients have AMD [5,10,12]. Most imaging techniques that are currently utilized by ophthalmologists, such as OCT, FP, and FA, give a clear view of the blood vessels within the retina, which makes it easier for physicians to determine if new blood vessels have formed within their patients [8,17]. Additionally, neovascularization was most likely the most reported symptom for diagnosing AMD as it is one of the more severe indications of the disease. Neovascularization was easily identifiable because it is clearly visible in imaging modalities such as FA. The new blood vessels that are formed from neovascularization can lead to an accumulation of fluid and even red blood cells within the retina [8,9]. This accumulation of substances can eventually lead to a loss of vision within a patient, which is why patients who present with neovascularization have typically already experienced a partial loss of vision. The severity of this sign is an additional factor that makes it easier for physicians to detect when they are attempting to determine if their patients have AMD. 

**Table 1:** The Usage of Imaging Technology in the Diagnosis of Age-Related Macular Degeneration OCT: optical coherence tomography; CFP: color fundus retinal photography; FA: fluorescein angiography; FP: fundus photography; FAF: fundus autofluorescence; NIR: near-infrared reflectance; OCTA: optical coherence tomography angiography; ICG: indocyanine green angiography.

Author (year)	Imaging techniques utilized in the study	Vascular ophthalmic issues	Non-vascular ophthalmic issues	Previous medical or ocular conditions	Sample size (patients)
Bellocq (2018) [6]	OCT	Neovascularization	N/A	Myopia	21
Cheung (2018) [7]	OCT, CFP	Neovascularization, hemorrhage	Drusen volume, geographic atrophy, hyperpigmentation	Diabetic retinopathy	4512
Cruz (2018) [9]	OCT	Hemorrhage	N/A	Diabetic retinopathy	2
Fan (2018) [8]	OCT, FA, CFP	Neovascularization, ischemia	Subfoveal choroidal thickness, macular atrophy	Macular atrophy	52
Freiberg (2018) [10]	OCT, FA, CFP	N/A	N/A	Diabetes/hypertension	230
Guymer (2019) [11]	OCT, FA	Hemorrhage, neovascularization	Drusen, RPE pigmentary abnormalities, drusen associated atrophy	Smoking	292
Hallak (2019) [12]	OCT	Hemorrhage, neovascularization	Drusen area/reflectivity	Diabetic retinopathy	686 eyes
Hsu (2020) [13]	OCT	Hemorrhage, neovascularization	N/A	Myopia	52
Keenan (2021) [14]	OCT	N/A	Drusen, geographic atrophy	Smoking	1127 eyes
Kertes (2020) [15]	OCT	Neovascularization, hemorrhage	Visual acuity changes	Diabetes	580
Khurana (2020) [16]	FA, OCT	Neovascularization	N/A	N/A	1097
Kim (2021) [17]	FP, FAF, NIR, OCT, OCTA, FA, and ICG	Neovascularization, ischemia	Soft drusen, reticular pseudodrusen, choroidal thickness	Hypertension/diabetes	661
Mitchell (2021) [18]	OCT	Hemorrhage, neovascularization	N/A	Diabetic retinopathy	271
Mukkamala (2020) [19]	OCT, FA	Hemorrhage	Geographic atrophy	Myopia	15
Nassisi (2019) [20]	OCT	Neovascularization	Drusen volume, subretinal drusen deposits, photoreceptor atrophy	Diabetic retinopathy	501
Oner (2018) [21]	OCT, FFA, FAF	Ischemia	N/A	Cataracts	8
Pan (2020) [22]	OCT	Neovascularization, hemorrhage	Pigment epithelial detachment	Diabetic retinopathy	38
Parravano (2019) [23]	OCT	Neovascularization	N/A	Diabetes	30
Querques (2021) [24]	OCT, FAF	Neovascularization	Reticular pseudodrusen, outer nuclear layer thickening	Myopia	20
Schmidt-Erfurth (2018) [25]	OCT	Neovascularization	N/A	Diabetic retinopathy	307
Schmidt-Erfurth (2020) [26]	OCT	Neovascularization	N/A	Diabetic retinopathy	1097
Waldstein (2020) [27]	OCT	Neovascularization	Drusen volumes, hyperreflective foci volumes	N/A	259
Wykoff (2021) [28]	OCT	Neovascularization	Geographic atrophy	Diabetic retinopathy	818
Yellapragada (2022) [29]	FP	Neovascularization	Drusen, pigmentary changes, geographic atrophy	N/A	4757

Though there are multiple image findings associated with the diagnosis of AMD, the most common non-vascular finding in patients with AMD was the presence of drusen [11,12,14]. Drusen are extracellular yellow deposits seen on fundoscopic examination and OCT imaging that consist of lipids and proteins. These deposits typically occur between the RPE and Bruch’s membrane [14,20]. The relationship between drusen and the progression of AMD is not completely understood, and a causal relationship between drusen and AMD was not demonstrated in this literature review. Despite this, the presence of drusen is a hallmark of early AMD, and larger drusen are associated with an increased risk of advanced disease [20]. Since drusen are a hallmark of early disease, and they persist from the early to late stages of AMD, it is clear why they are such a common non-vascular finding in AMD [27]. As seen in Table 1, papers included in the review focused not only on the presence of drusen but specific drusen characteristics such as drusen area, volume, hyper-reflectivity, and whether the drusen were soft or hard [6-29]. Regardless of the different aspects of drusen being analyzed, the presence of drusen in eyes with AMD was common throughout the papers in this review.

Associated medical conditions

Among all of the studies analyzed in this review, based on the information presented in Table 1, diabetes mellitus (DM) with or without progressed diabetic retinopathy (DR) was by far the most prevalent previous medical condition noted in patients diagnosed with AMD [6-29]. The elevation of blood sugar levels and the subsequent increase of sorbitol within the body through the aldose reductase pathway are consequences of diabetes, leading to various issues like retinopathy. DR is generally considered to be a disease affecting the inner retina and is a complication observed in later-stage diabetic patients from damage to the blood vessels in the back of the eye. DM and its relation to the development of AMD have long been a topic of many studies and even greater debate. While several major reports have shown a positive correlation between AMD and DM, others have shown no such effect or even an inverse correlation [30]. Despite some contradictory studies, the growing consensus in the ophthalmological field is that there is some connection between DM and AMD. While DM and AMD are separate diseases that affect different parts of the retina, they do share some common etiology. Carbohydrate-related mechanisms have been linked to the pathogenesis of these diseases, with increased drusen seen in patients with glucose intolerance [31]. In addition to the carbohydrate-related mechanism, both wet AMD and DR are mediated by VEGF. The use of anti-VEGF pharmaceuticals has thus been a proven effective treatment for both diseases and further links the two together [32]. This implies that improving control over DM and limiting the progression to DR may also lessen the chances of developing AMD. However, far more large-scale longitudinal studies are needed to confirm this.

Further prospects and current limitations

In developed countries, AMD is the most common cause of blindness in people over the age of 65, and it presents in two major forms [33]. This review offers a comprehensive assessment of diagnosing AMD, covering all relevant aspects. First, the data gathered to provide insight into some of the illnesses and symptoms that might point to the risk or even occurrence of AMD in a patient. This can help guide physicians in their regular care of patients in knowing when to suspect and check for AMD. Similarly, having comprehensive knowledge of the symptoms that can present with AMD will also aid physicians in knowing the different treatments that need to be prescribed. In addition, this review takes a look at the various modalities utilized in the diagnosis of ocular disorders. One such modality examined was OCT, a test commonly used to check an individual's eyes for AMD. Our paper allows physicians to better understand the limitations of their current diagnostic methods for AMD, which may lead to a more holistic approach to the diagnosis and treatment of the disease. The effectiveness of several retinal imaging modalities for the diagnosis of vascular and non-vascular ocular disorders was successfully examined in this review. We were able to identify the imaging modalities employed in the diagnosis of ocular disorders by analysis of 24 papers. One of the reviews' weaknesses was the lack of diversity for other imaging modalities, such as ultrasound or retinal imaging using Optomap, and the fact that the majority of the studies that were found were on OCT and FA. Future research could therefore compare different imaging modalities and broaden the variety of imaging modalities. This would give a good understanding of whether there are more effective ways to diagnose vascular and non-vascular retinal disorders. The lack of data needed for it to be statistically significant was another drawback. Additionally, there remains a necessity for enhanced technology as the current imaging tools possess limitations in their capacity to offer a complete depiction of the ailments and issues impacting the retina. Some limitations of eye imaging technologies include factors like the quality of images being affected by media opacities, motion artifacts, and image processing. Additionally, fundus imaging may produce false colors, and FA can be invasive while providing a poor view of choroidal vasculature. Other drawbacks include lower resolution (10 µm), poor resolution of retinal layers, slower image capture, and the lack of an eye-tracking feature in OCT [34]. As a result, an improved imaging technique and technology will help in developing a better diagnosis and treatment strategy. The best imaging modality with the highest rate of accurate diagnosis and, consequently, the greatest treatment options may have been found by looking at the patient outcomes. Analyzing post-diagnostic and post-treatment outcomes will provide a more clear understanding [35]. Future research on patient outcomes should therefore make it possible to accomplish that. These factors should be taken into account when examining the influence of various imaging modalities on the diagnosis and therapy of vascular and non-vascular ocular disorders. Ophthalmologists commonly use OCT, FP, and FA imaging techniques in their practice. It would be beneficial for more studies to incorporate additional imaging techniques like ultrasound, Optomap, CFP, and FFA. One area that requires improvement in investigations is imaging techniques. The technology used for imaging needs to be enhanced for more precise and quicker diagnoses. This would allow ophthalmologists to detect more signs among patients who have AMD and a way to check for signs early on. It would be beneficial for the study to enhance its reporting of the patient's outcome and the long-term impact of the imaging. Other factors to consider with imaging techniques would be to track and report the effect of the imaging techniques in different stages of AMD. Providing a larger sample size with greater variety would produce more accurate results. Examining all the studies showed that DM with or without progressing DR were the most previous medical conditions noted in patients diagnosed with AMD. To properly evaluate various AMD patient populations, the study should also include a larger sample of patients who are not diabetic. It would be beneficial to have a more diverse patient population with varying ethnicities and backgrounds to assess how imaging impacts different groups.

## Conclusions

Diagnosis of AMD requires the use of imaging technology to investigate and understand the pathophysiology of the retina. This review attempts to analyze the effectiveness of OCT and other imaging technologies in detecting vascular and non-vascular ophthalmic issues that are caused by AMD. Using data collected from the review a statistical analysis was undertaken, and the results revealed that the combination of OCT and other imaging technologies increased the detection of ocular difficulties caused by AMD. This suggests that multiple forms of imaging technology are necessary to obtain a more clear assessment of a patient’s AMD condition. Further research is necessary to truly validate these claims as there were some limitations to the data and the methodology that was utilized. 
